# Fast Temporal Graph Convolutional Model for Skeleton-Based Action Recognition

**DOI:** 10.3390/s22197117

**Published:** 2022-09-20

**Authors:** Mihai Nan, Adina Magda Florea

**Affiliations:** Faculty of Automatic Control and Computers, Politehnica University of Bucharest, 060042 Bucharest, Romania

**Keywords:** action recognition, temporal convolutional network, sequence modeling, fast convolutional model, graph convolutional network, data augmentation

## Abstract

Human action recognition has a wide range of applications, including Ambient Intelligence systems and user assistance. Starting from the recognized actions performed by the user, a better human–computer interaction can be achieved, and improved assistance can be provided by social robots in real-time scenarios. In this context, the performance of the prediction system is a key aspect. The purpose of this paper is to introduce a neural network approach based on various types of convolutional layers that can achieve a good performance in recognizing actions but with a high inference speed. The experimental results show that our solution, based on a combination of graph convolutional networks (GCN) and temporal convolutional networks (TCN), is a suitable approach that reaches the proposed goal. In addition to the neural network model, we design a pipeline that contains two stages for obtaining relevant geometric features, data augmentation and data preprocessing, also contributing to an increased performance.

## 1. Introduction

Human action recognition (HAR) is a continuous problem, unlike people detection or face recognition, which are based on static bounding box detection. This aspect along with the variation in the duration and in the way the user performs the actions makes this problem a complex task. Thus, for an approach based on artificial neural networks, a great deal of information is needed to train a model capable of generalizing the characteristics of each action. Another important aspect is that we can describe an action using two relevant components: a temporal dimension and a spatial dimension. Temporal convolutional networks (TCNs) are one of the most effective methods for modeling vast types of sequences, showing promising results in multiple fields, such as Computer Vision, Natural Language Processing, and Forecasting. Being a subtype of a classical convolutional network, TCNs are better suited for learning spatial and temporal features, often making this more efficient than recurrent neural networks (RNNs). Another helpful feature of TCNs is their very flexible architecture. For this type of neural layer, we can configure the temporal window properties using different kernel sizes, dilations, and strides. In addition, these TCN-type units contain a small number of parameters and can easily integrate residual connections.

In this article, we explore the skeleton-based approach to HAR, highlighting the advantages of it compared to those based on other methods, such as RGB images or depth maps. These advantages allowed us to develop a solution capable of recognizing human actions in various environments with minimal inference time. Because the human skeleton is a graph, we can successfully apply graph convolutional networks (GCNs) in neural models that solve this human action recognition task. We can use GCN-type layers for various tasks, such as node classification, link prediction, clustering, or network similarity. Besides these independent tasks, GCNs present the property of extracting relevant features from a spatial perspective because these layers apply operations directly on graphs and take advantage of the structural information. In this article, we highlight the property of a GCN to project handcrafted geometrical features into a high-dimensional space but also how we can combine the properties of GCNs and TCNs to obtain a fast and performant neural model for skeleton-based action recognition.

We consider pro-activity one of the key properties that a robot must fulfill to be named a social robot. And as a robot cannot be called pro-active if it fails to identify the actions performed by those around it, we implicitly come to the conclusion that the human action recognition module represents a key concept in the development of a social robot. The development of such a module is very challenging because the solution must be able to provide a prediction in real time. Additionally, it is indicated that the neural model has a small number of parameters that do not require a large amount of memory to store the weights. The pipeline proposed by us in this article takes into account these two essential aspects that a human action recognition module must fulfill to be successfully integrated into a robotic platform. Moreover, we remedy an additional limitation through the proposed approach. We know that the performance of neural models depends on the data used in the training process. Most of the datasets proposed for this task are collected using fixed cameras with clear characteristics and a limited number of participating subjects. Through the augmentation transforms introduced in our pipeline, we set out to solve this problem.

Our contribution to this paper contains:

1. We propose an efficient pipeline for solving the problem of human action recognition using skeletal data. This pipeline consists of two important modules: a data processing module and a module that contains a neural model that classifies human actions.
2. We design a small neural model, based on various types of convolutional layers, that achieves good accuracy and a high inference speed.
3. We perform an extensive analysis of the results achieved by the proposed neural model to validate its robustness. The purpose of these experiments and analyses is to show the potential of the proposed method in comparison with the existing solutions.

In Section 2, we included some high-performance methods proposed in the literature for the human action recognition problem, starting from skeletal data. These models represent a source of inspiration for the proposed approach. We detailed the proposed approach and highlighted the structure of the neural model in Section 3. The applied preprocessing operations and augmentation transforms are also presented in Section 3. To evaluate the proposed method, we chose a benchmark applied for such solutions and presented the results achieved by the model for this dataset in Section 4. Finally, in Section 5, we presented the conclusions of this research work and outlined further development directions.

## 2. Related Work

The previous research [1,2,3] highlights that to recognize actions, the human brain analyzes two levels of visual processing: one high level and one low level. The high-level processing deals with semantic processing and is based on global features, and the low-level processing is focused on kinematic features. As the methods based on Deep Learning try to simulate the behavior of the human brain, their development takes into account the theoretical aspects discovered experimentally in various studies. Regarding the approaches based on skeletal representations, the emphasis is placed on the low-level processing that analyzes the kinematic characteristics.

Wang et al. [4] started from the limitation of the skeletal representation in determining semantic information and proposed temporal–channel aggregation graph convolutional networks (TCA-GCN). This neural unit consists of two specialized components in the combined feature extraction at the temporal and spatial levels. The temporal aggregation module is aimed at determining the relevant templates for each action at a temporal level. The channel aggregation module combines the features that are determined based on the topology of the human skeleton with those related to the dynamics of the skeleton. Moreover, it has the property of combining spatial features from several channels. This method achieves a good performance on the NTU RGB+D benchmark, but it has a considerably higher number of weights than the solution proposed by us. This fact also determines a lower inference speed than that of our model introduced in this article. For this reason, it is complicated to use such a neural network for a practical problem for which a real-time prediction using limited hardware resources must be provided.

Chen et al. [5] tried to design a method of modifying GCN-type layers that would reduce the difficulty of modeling channel-wise topologies. The proposed name for this technique is channel-wise topology refinement graph convolution (CTR-GC) and contains three stages: channel-wise topology modeling, feature transformation, and channel-wise aggregation. In the first stage, a trainable shared topology is improved by channel-wise topology modeling, using inferred channel-specific correlations. The second stage is the one that transforms the input into a high-dimensional representation. These first two stages are applied independently, and the last step is the one that combines the results of the first two to determine the output using channel-wise aggregation. Starting from this module, the authors designed a neural model specialized in skeleton-based human actions recognition. Unlike previous approaches, they used the entire graph, describing the human skeleton for the neighborhood of each joint.

Xie et al. [6] proposed a new network design described as a cross-channel graph convolutional network. This significantly improves the ability of the model to capture local features among adjacent vertices. This came as a result of their observation that other previous models may not extract the local features of adjacent vertices in the graph effectively. They further improved this design by adding a channel attention mechanism which filters out the features that are unrelated to the action being observed. With this novel approach, local features strongly related to the observed action can be more efficiently captured, and more accurate deeper features can be computed. Models using this approach can thus be more robust and can obtain higher accuracy scores. This was shown for the above-mentioned model trained on both the NTU-RGB+D [7,8] dataset, with a score of 95.33% in Crossview, and the Kinetics-Skeleton dataset, with a 58.9% Top 5 accuracy score.

Song et al. [9] addressed the problem of approaches based on increasingly deeper neural networks. These neural models have too many parameters, and their training becomes complicated for datasets with a large number of actions. In addition, even after training, such approaches have the disadvantage of a too long inference time, which makes them inapplicable for frameworks that require real-time predictions. In their approach, Song et al. propose a fast and efficient neural model based on the concept of graph convolutional networks. For this model, the authors performed a series of experiments in which they demonstrated that the proposed model achieved the best performance when using three branches for the features extracted from the data predicted by Kinect. The authors proposed a module based on a GCN containing a residual connection called a residual GCN (ResGCN). This module consists of one spatial and one temporal block applied in sequential order.

Song et al. [10] observed that most models that aim to extract discriminative features over all skeletal joints to solve the skeleton-based action recognition problem tend to suffer from being overly complex and over-parametrized. In the proposed solution for this problem, they started with a Multiple Input Branches network topology and constructed a graph convolutional network baseline architecture that can vary its width and depth through a compound scaling strategy. This allowed them to create a family of efficient GCN baselines with high accuracies and fewer trainable parameters, aptly named EfficientGCN-Bx, where x is the scaling parameter.

Approaches based on GCNs have achieved a good performance for the HAR problem on several benchmarks containing skeleton representation [11,12,13,14,15]. Unfortunately, these layers have a limitation: they do not differentiate between the importance of each edge. The role of each connection between joints from the graph that describes the human posture can differ from one activity to another. For example, the edges between arm joints are more relevant than those between the joints on the legs for the actions that involve hands (e.g., “reading” and “writing”). Lee et al. addressed this disadvantage in [16], proposing a hierarchically decomposed graph convolutional network (HD-GCN). The first step applied by this new proposed neural unit consists of decomposing the graph into a rooted tree that has the Center of Mass as its root. The second applied operation is the Fully Connected Inter Hierarchy Edges which start from the decomposed graph and identify the relationships between the major distant joint nodes. To ensure the training stability, the authors proposed the normalization of the obtained adjacency matrices. It is important to note that they chose to leave all matrix elements in the form of the weights that are learned during the training process. Unlike this approach, we use two variants of graphs in the proposed solution. We propose the first version of the graph to determine some relevant features at the spatial level. We compute the adjacency matrix for this graph, considering a maximum distance equal to 2. The second version of the graph used by us is one in which the neighborhood of each node contains all the other nodes.

The solution proposed by Trivedi and Sarvadevabhatla [17] emphasizes efficiency and generalization. Through the proposed experiments, they demonstrated that the PSUMNet-based approach obtains state-of-the-art results for the Cross-Subject protocol of the NTU RGB+D 120 dataset. Moreover, their solution has a much smaller number of weights than other approaches with similar results. They demonstrated generalizability from the perspective of the representation used. Thus, they tested the proposed neural network from the perspective of three representations: the sparse skeleton (the classic skeleton representation predicted by the Microsoft Kinect sensor), the part-based skeleton (contains 22 points of articulation that describe the fingers), and the dense skeleton (a combination of the first two). The method based on the Part-Streams-Unified Modality contains three branches: one that analyzes the entire skeleton, one that computes predictions based on the joints of the hands, and one that predicts the scores from the legs perspective. They trained each branch independently, and finally, a fusion mechanism was applied between the predicted scores. This method is the one that obtains the best results for the NTU RGB+D benchmark, but it is based on a neural network with 2.8 M parameters, while the network proposed in our approach is also a multi-branch one, but which contains only 1.58 M parameters.

We previously proposed in the paper [18] several methods of modeling a sequence of frames describing a skeleton-based action. One of these methods allows the application of 3D convolutional layers. This method uses the idea of rearranging the 25 joints in a 5 × 5 matrix in which we located the incident points in adjacent cells. We integrated these methods into neural architectures based on temporal convolutional network (TCN) or recurrent neural network (RNN) layers. The experimental results demonstrated that the RNN-based approach has a higher inference speed, but the TCN-based approach achieves a better performance for the NTU RGB+D [7,8] benchmark.

Starting from these existing ideas and approaches, we have developed a method based on various types of convolutional layers that presents an increased inference speed and performance comparable to the existing ones for the NTU RGB+D dataset [7]. Moreover, the proposed neural model has a small number of parameters, which makes it easy to train. Moreover, we can integrate this action classifier in robotic frameworks that run in real time using limited hardware resources. The neural model contains layers of the CTR-GC type proposed by Chen et al. and uses ideas from our previous approach [18]. We determined the handcrafted geometrical features used to train the model using the formulas proposed by Song et al. in [9].

## 3. Proposed Method

In this section, we present the approach proposed for the problem of human action recognition, which consists of a preprocessing stage and a neural model based on various types of convolutions layers. This approach represents an improvement to the previous method proposed by us in the paper [18].

### 3.1. Data Preprocessing

Given that human action recognition is a problem of classifying a temporal sequence, we propose a method based on the most used types of neural networks for such problems: TCNs and GCNs. The data used by us in the proposed approach are in skeletal form. We represent each action as a tensor T×25×3×M, where *T* represents the number of frames, and *M* represents the number of skeletons. In the skeleton modality from the NTU RGB+D dataset, we have a set containing 25 joints for each skeleton, and we know 3 coordinates for each joint. Starting from these values, we encode each sample in a tensor with *T* = 300 and *M* = 2. Using this tensor, we extracted 3 categories of information, starting from the approach proposed by Song et al. [9].

**Figure 1:**
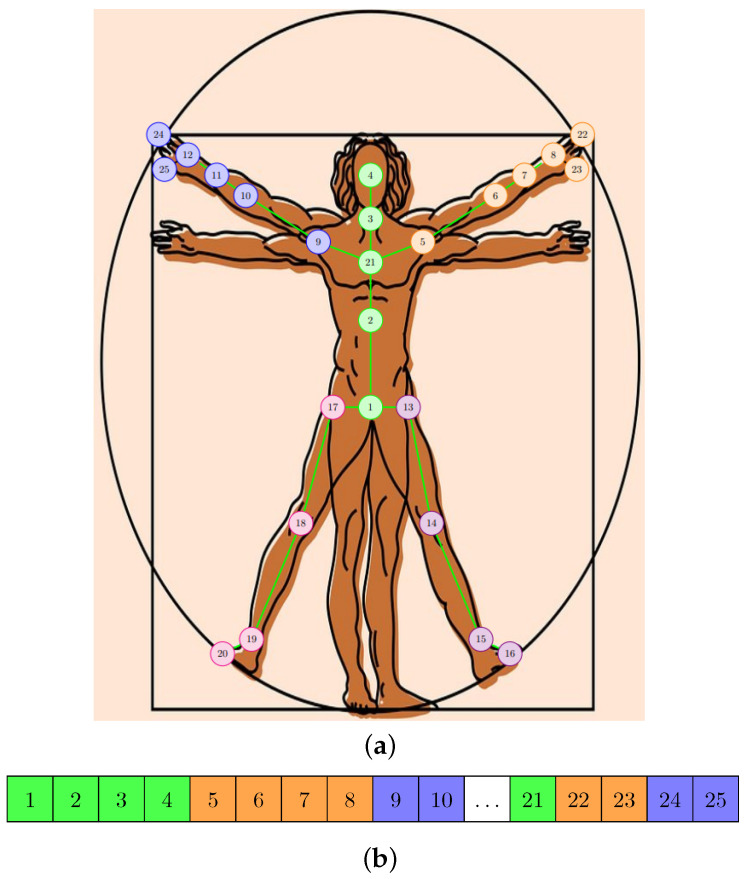
The representations used for the human skeleton in the proposed approach. (**a**) The graph composed of the points of articulation. (**b**) Linear representation of joints.

In Figure 1, we included the sequence modeling methods that we used in our approach: graph form (Figure 1a) for GCN-type layers and linear form (Figure 1b) for layers of TCN type. The connections that appear between the joints in the graph structure were also used to determine the geometric features. The current method starts from the same categories of features, but by introducing some optimizations at the level of the structure of the neural model and by applying augmentation techniques, it improves the inference speed, increases the accuracy achieved by the model, and reduces the number of parameters.

The first category of features is based on the coordinates of the joints. We use the following formulas for the **joint-branch**:

(1) $$joint\_features_{j_{i}}^{t}=\left(x_{j_{i}}^{t},y_{j_{i}}^{t},z_{j_{i}}^{t},x_{j_{i}}^{t}-x_{j_{c}}^{t},y_{j_{i}}^{t}-y_{j_{c}}^{t},z_{j_{i}}^{t}-z_{j_{c}}^{t}\right),\forall t\in \left\{1,2,\cdots ,T\right\}$$

where $j_{i}$ represents a joint (i∈{1,2,⋯,25}) and $j_{c}$ is the point we considered to be an approximation of the center of gravity of the skeleton. In the proposed method, we used c=1. The coordinates predicted by Kinect for each joint $j_{i}$ in $R^{3}$ space are $x_{j_{i}}$, $y_{j_{i}}$, and $z_{j_{i}}$.

For the second category of features, we determined information that describes the speed with which the subject performs the action. We use the following formulas for the **velocity-branch**:

(2) $$\begin{array}{c} velocity\_features_{j_{i}}^{t}=\left(x_{j_{i}}^{t+2}-x_{j_{i}}^{t},y_{j_{i}}^{t+2}-y_{j_{i}}^{t},z_{j_{i}}^{t+2}-z_{j_{i}}^{t},x_{j_{i}}^{t+1}-x_{j_{i}}^{t},y_{j_{i}}^{t+1}-y_{j_{i}}^{t},z_{j_{i}}^{t+1}-z_{j_{i}}^{t}\right),\\ \forall i\in \{1,2,\cdots ,25\},\forall t\in \{1,2,\cdots T-2\} \end{array}$$

The last category contains features that describe the bones. We use the following formulas for the **bone-branch**:

(3) $$\begin{array}{c} bone\_features_{\left(j_{u},j_{v}\right)}^{t}=\left(x_{j_{u}}^{t}-x_{j_{v}}^{t},y_{j_{u}}^{t}-y_{j_{v}}^{t},z_{j_{u}}^{t}-z_{j_{v}}^{t},a_{(j_{u},j_{v}),x}^{t},a_{(j_{u},j_{v}),y}^{t},a_{(j_{u},j_{v}),z}^{t}\right),\\ \forall \left(j_{u},j_{v}\right)\in \left\{1,2,\cdots 25\right\}\times \{2,21,21,3,21,5,6,7,21,9,10,11,1,13,14,15,1,17,18,19,\\ 23,8,25,12\},\forall t\in \{1,2,\cdots ,T\} \end{array}$$

where joints $j_{u}$ and $j_{v}$ are adjacent, and $l_{(j_{u},j_{v}),x}=x_{j_{u}}-x_{j_{v}},l_{(j_{u},j_{v}),y}=y_{j_{u}}-y_{j_{v}},l_{(j_{u},j_{v}),z}=z_{j_{u}}-z_{j_{v}}$, and $a_{(j_{u},j_{v}),x}^{t},a_{(j_{u},j_{v}),y}^{t},a_{(j_{u},j_{v}),z}^{t}$ are angle values calculated using the following formulas:

(4) $$\begin{array}{c} value_{x}=\frac{l_{(j_{u},j_{v}),x}}{\sqrt{l_{(j_{u},j_{v}),x}^{2}+l_{(j_{u},j_{v}),y}^{2}+l_{(j_{u},j_{v}),z}^{2}}},value_{y}=\frac{l_{(j_{u},j_{v}),y}}{\sqrt{l_{(j_{u},j_{v}),x}^{2}+l_{(j_{u},j_{v}),y}^{2}+l_{(j_{u},j_{v}),z}^{2}}}\\ a_{(j_{u},j_{v}),x}=\arccos\left(value_{x}\right),a_{(j_{u},j_{v}),y}=\arccos\left(value_{y}\right)\\ value_{z}=\frac{l_{(j_{u},j_{v}),z}}{\sqrt{l_{(j_{u},j_{v}),x}^{2}+l_{(j_{u},j_{v}),y}^{2}+l_{(j_{u},j_{v}),z}^{2}}},a_{(j_{u},j_{v}),z}=\arccos\left(value_{z}\right) \end{array}$$

### 3.2. Data Augmentation

Neural networks with convolutional layers require a large amount of information in the training process to avoid the overfitting issue. Noticing that the proposed neural model reaches over 90% accuracy for the training set after only 10 epochs, we decided to artificially generate new samples using various types of transforms applied to the *X*, *Y*, and *Z* coordinates provided in the dataset. The transforms we applied in the proposed approach are: uniform sample, random move, and random rotation. We applied these transforms to each step of the training process.

The first applied transformation, which we call uniform sample, is the one in which we randomly extract a series of frames from a sample according to a fixed size. More precisely, this operation transforms a sample with the shape C×T×V×M into a sample of the type $C\times T^{\prime }\times V\times M$, where *C* represents the number of channels, *T* represents the initial number of frames, $T^{\prime }$ is the number of frames in the new resulting sample, *V* is used for the number of joints, and *M* means the number of skeletons. For this transformation, we distinguish two situations: if the number of frames in the sample is greater than the fixed size or if the temporal dimension of the action is smaller than the chosen temporal window. If we need to reduce the number of frames, we determine the number of intervals from which we can select these frames and the size for each one using Equation (5). From each such range, we randomly select a frame.

(5) $$interval\_size=\frac{T}{T^{\prime }}\;number\_of\_intervals=T^{\prime }$$

The application of this transformation presents two advantages: by reducing the number of frames, we can eliminate redundant information, which determines the optimization of the model from the calculability perspective, and we can simulate that the actions are collected using sensors with different frame rates.

We applied the transformation that randomly moves the skeleton to simulate the fact that the person is in different positions during data collection. This augmentation can be helpful if the pre-trained neural model is used for a social robot, as it will be in constant motion.

We randomly chose three values from the interval [−θ,+θ] for each sample to apply the rotation. We generated three values because we wanted to apply the rotation transform on all three coordinate axes. We applied this rotation operation frame by frame. Such augmentation simulates the fact that we would have various angles at which the Kinect sensor would be positioned, while in reality, there exists only three angles for the NTU RGB+D dataset.

### 3.3. Temporal Graph Convolutional Model

**Figure 2:**
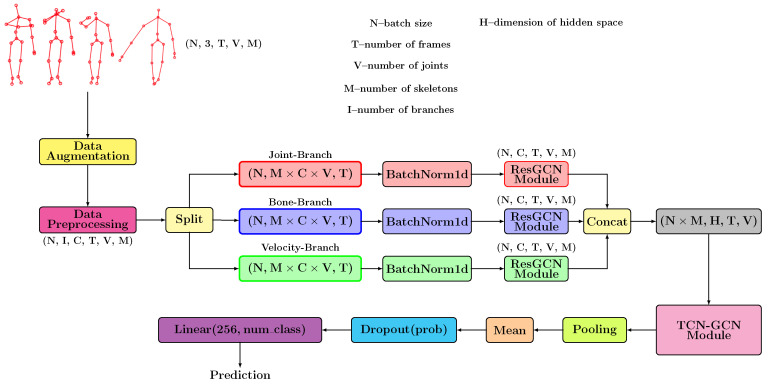
The pipeline proposed consists of two fundamental components: the data processing stage and the convolutional model.

The complete pipeline designed for our solution is presented in Figure 2. It contains two stages: a stage for calculating the features and a stage for applying the proposed neural model.

Initially, we start from a tensor with size N×3×T×V×M for which we apply a series of augmentation transformations. After these operations, we perform the data preprocessing in which the features are determined based on the geometric formulas presented in Section 3.1. Next, we project these features in a high-dimensional space using some neural layers. We perform the extraction of additional features independently for each branch using, in the first phase, a Batch Normalization-type layer followed by a series of ResGCN-type layers. In Section 3.4, we described in detail the architecture of this module, highlighting its importance. We designed the TCN-GCN module based on a series of GCN-TCN-type units that aim to determine the temporal characteristics and extract the spatial patterns. Section 3.5 is the one that presents the structure of this module. These units keep the spatial size, but those that use stride will halve the number of frames after application. We preserve the spatial dimension to further apply the GCN-type layers that use the graph described by the human skeleton. The TCN-GCN module analyzes the features from a spatio-temporal point of view, resulting in a tensor with $N\times M\times 256\times \frac{T}{8}\times V$ shape. We apply an Adaptive Average Pooling-type layer to obtain a final prediction that summarizes all the $\frac{T}{8}$ frames and all the *V* joints. The result of applying this layer is a tensor with dimension N×M×256. We calculate an average of the features determined for the two skeletons to obtain a result invariant to the order of the skeletons. The proposed model predicts the classification probabilities using a fully connected layer.

### 3.4. ResGCN Module

**Figure 3:**
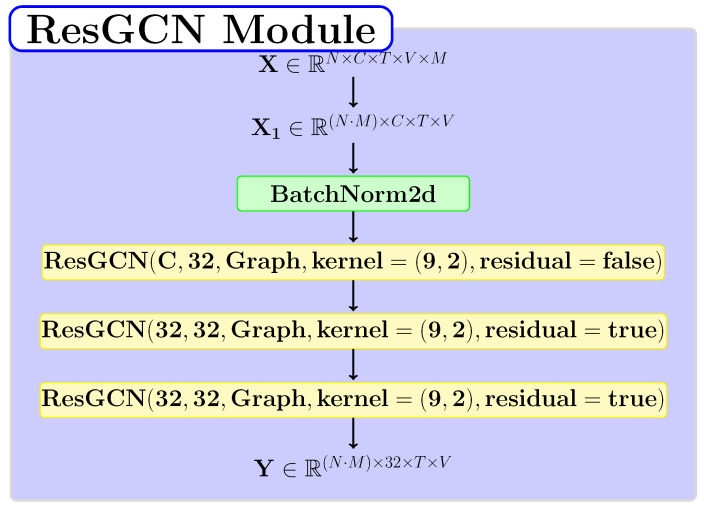
The structure of the ResGCN layer-based module.

In the pipeline proposed within our method, we independently use a module based on ResGCN blocks to extract spatial or temporal dependencies from the data corresponding to each branch. In Figure 3, we highlight the proposed structure for this module. The tensor provided as input has the number of channels C=6, and the module reshapes it to be applied independently for each skeleton. Thus, the batch size becomes equal to N·M, where *N* represents the initial batch size, and *M* is the number of skeletons.

**Figure 4:**
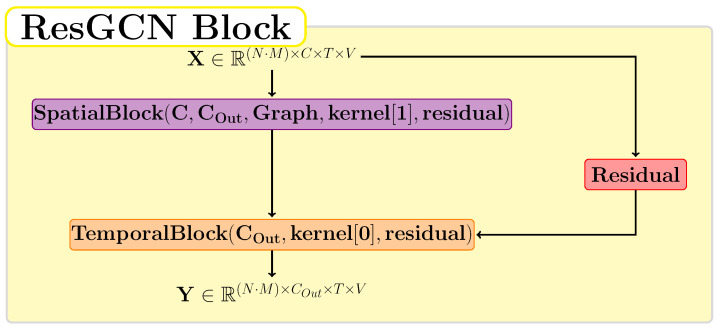
The architecture used for a ResGCN block in which the residual block consists of $f_{1}\left(X\right)=X$ or $f_{2}\left(X\right)=0$.

Figure 4 shows the structure of a ResGCN block. This unit is the one proposed by Song et al. [9] and contains a spatial block, a temporal block, and a residual connection. If the value of the residual parameter is true, then the residual layer is implemented in the form of the function $f_{1}\left(X\right)=X$. Otherwise, we use the function $f_{2}\left(X\right)=0$ for the residual layer. This unit presents the property of preserving the spatial and temporal dimensions. The value of kernel[1] represents the size of the neighborhood of a graph node, and kernel[0] is the size of the temporal window. We used the value 2 for the number of neighbors in a node and the value 9 for the temporal window.

The graph used for these ResGCN-type units is one in which the maximum graph distance is set to 2. The bottleneck structure used for the spatial and temporal blocks ensures a model with high inference speed and which requires a low number of epochs for training. Moreover, the module presents an additional optimization, obtained by changing the batch size and applying operations in parallel for the two skeletons.

### 3.5. TCN-GCN Module

**Figure 5:**
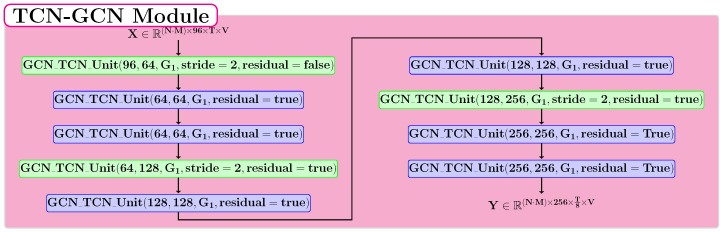
The proposed structure for the TCN-GCN module.

The spatio-temporal module represents the fundamental part of the proposed neural model, and its structure is described in Figure 5. The basic unit of this module is the GCN-TCN block proposed by Chen et al. [5]. This block is composed of two of the most relevant types of neural layers for the human action recognition problem: GCN and TCN. In our approach, we used a graph in which the neighborhood of each node contains the entire skeleton graph for these blocks. This module consists of 9 layers applied sequentially, and each one is a GCN-TCN Unit. Three of these layers halve the temporal dimension by using a stride value of 2. The blue colored layers in Figure 5 preserve the temporal dimension.

**Figure 6:**
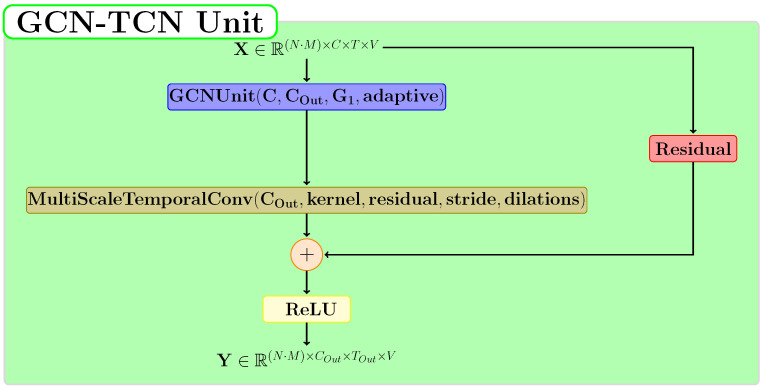
The proposed structure for the TCN-GCN module.

Figure 6 presents the architecture of the TCN-GCN Unit. For the GCN layer, we used the *true* value for the adaptive parameter. The temporal window size for the MultiScaleTemporalConv layer is set to 5 in our experiments. This layer applies two dilations: the first with a value of 1 and the second with a value of 2. For the residual block, we have three possible options: function $f_{2}\left(X\right)=0$ if the residual link is not applied, function $f_{1}\left(X\right)=X$ when the residual parameter is true and *C* = *C*1, and, otherwise, a TCN-type layer.

## 4. Results

### 4.1. NTU RGB+D Dataset

NTU RGB+D [7,8] is a dataset that contains two versions: the first proposed version contains 60 classes, and the second version added another 60 classes to those in the first version. The actions in this dataset are actions that are performed by one or two people. In the current paper, we exploit the version with 60 classes to validate the proposed model. All 60 types of actions are performed indoors. The actions included in this dataset can be divided into three main categories: daily actions, mutual actions, and health-related actions.

This dataset was recorded using three cameras positioned at the same height but placed at different horizontal angles: $-45^{\circ }$, $0^{\circ }$, and $+45^{\circ }$. Each subject who participated performed each action twice: once toward the left camera, and once toward the right camera. Each sample was collected using a Microsoft Kinect v2, RGB images, depth maps, skeleton data (3D locations of 25 major body joints), and infrared images.

The authors proposed two test protocols for the first version of the dataset: Cross-Subject and Cross-View. The Cross-Subject protocol is more complex because they distributed the samples in the training and test subsets according to the subjects. This aspect produces greater diversity because each subject acts characteristically. In the Cross-View protocol, the authors proposed to build the two subsets according to the camera that recorded them. Thus, we used samples from two cameras for training and the samples collected with the third camera for testing.

The actions included in the two versions of the dataset are actions performed by a single person or by two people. The first version of the dataset includes 40 daily actions, such as: *drinking*, *eating*, *reading*, *writing*, and *brushing teeth*; 9 actions related to health: *sneeze/cough*, *staggering*, *falling*, *touch head (headache)*, *touch chest (stomachache/heart pain)*, *touch back (backache)*, *touch neck (neckache)*, *nausea or vomiting condition*, and *use a fan (with hand or paper)/feeling warm*; and 11 mutual actions performed by two people: *punching/slapping another person*, *kicking another person*, *pushing another person*, etc. The second version of the dataset introduces 42 daily actions, 3 health-related actions, and 15 mutual actions.

### 4.2. Implementation Details

In our experiments, we obtained the best results for a training process with 70 epochs. The optimizer used to train the proposed neural model was the SGD. For this optimizer, we used the Nesterov momentum and the following values for the hyperparameters: learning_rate = 0.1, weight_decay = 0.0002, and momentum = 0.9. Regarding the learning rate, we tested several variants, but the network achieved the best performance using a Cosine scheduler with a warm-up strategy. The size of the batch used for training and testing is 64. For the Dropout-type layer, we chose the probability equal to 0.3.

### 4.3. Ablation Study

In this section, we analyze the proposed pipeline from the perspective of its configuration and the hyperparameter values. We performed the experiments included in this section for the Cross-Subject protocol (v1—60).

Initially, we checked the influence of the interval from which we chose a random value for the rotation angle. We tried three variants and included the results obtained in Table 1. The results are almost identical from the perspective of the Top 5 accuracy. Instead, for the higher values of θ, the Top 1 accuracy reached by the model decreases.

To discover the importance of the augmentation transformation, we carried out a series of experiments for which we included the obtained performances in Table 2. We also highlighted the importance of the Dropout layer used for regularization. In these experiments, we kept the θ value constant. We used the SGD optimizer and 70 epochs for the training process for all the runs. It is relevant to note that when we do not use augmentation transformations, we obtain a similar accuracy but a much lower inference speed. Thus, we can conclude that by applying the Uniform sample augmentation operation, we eliminate redundant information, but through the rest of the transformations, we reduce the loss of the relevant features. In conclusion, by applying the augmentation process, we obtain a model that achieves a similar performance but is much faster and computationally efficient.

We analyzed how the temporal and spatial kernels influence the model performance. For this, we proposed a series of experiments, presented in Table 3. From the obtained results, we can see that there are no significant differences between the performances. We note that the experiment that obtained the best accuracy for the Cross-Subject is the one in which we used for ResGCN temporal-window = 11 and spatial-window = 3, respectively, and temporal-window = 5 for TCN. Instead, this experiment is the one for which we obtained the lowest inference speed. We can conclude that from the obtained results, we can see that there are no significant differences between the performances.

### 4.4. Experimental Results

We used both versions of the NTU RGB+D dataset [7,8] with all proposed protocols to test the proposed model. The obtained experimental results demonstrate that the proposed model achieves similar performances to the state of the art, even if it presents a smaller number of parameters. The conducted experiments highlight that the inference time obtained for the proposed pipeline is better than the rest of the methods. Therefore, the training process of such a model takes less time.

In Table 4, we included the performance of our architecture and some results reported in the literature for the first version of the NTU RGB+D benchmark [7]. For each model, we included the model size (the number of parameters) and the accuracy obtained for the Cross-Subject protocol and that for the Cross-View protocol. Three architectures have a smaller number of parameters than the proposed one, but only two of them obtain better results than our method: PA-ResGCN-N51 [9] and CTR-GCN [5]. Starting from this observation, we decided to make a comparison between these three from the perspective of the inference time.

Other methods exist in the specialized literature that obtained a better score than our method for the two test protocols, but these methods are based on neural networks with a larger number of weights. For this reason, these models are more difficult to train, have a longer inference time, and require hardware equipment with additional resources. For example, NAS-GCN [26] is a neural model that achieves similar performance to the method proposed by us but has a size 4 times larger.

We selected the best combination of the hyperparameters and evaluated it from the perspective of the four protocols proposed for the NTU RGB+D dataset. We included these results in Table 5 and Table 6. In these tables, we highlight the results obtained for each protocol from the perspective of two metrics:

- Top 1—checking only the action for which the neural model predicted the highest value.
- Top 5—checking if any of the first five actions predicted by the network represent the correct action.

These results demonstrate that the proposed model has performant Top 5 accuracy. For all the analyzed protocols, the Top 5 accuracy has a value higher than 97%. To understand why this difference appears between the Top 1 and Top 5 accuracy, in Section 4.5, we have included a detailed analysis of these results. We performed this analysis from the perspective of the classes with best accuracy and those with the weakest accuracy achieved by the proposed model. To obtain these results, we trained the proposed model for 70 epochs. We also applied all the augmentation transforms described in Section 3.2. Thus, we randomly extracted 200 frames at each step, considering the sample size. The value used for the probability of the Dropout layer is equal to 0.3. The value of θ used to randomly choose the angle for rotation is equal to 0.3 in these experiments. The batch size used for these experiments is 64.

In the case of the extended version of the dataset, we obtain a significant difference between the Top 1 and Top 5 accuracy. We can explain this greater difference based on the large number of similar classes contained in the NTU RGB+D 120 dataset. It is important to note that the difference is almost 12 in the case of the Cross-Subject protocol (v2—120). This protocol is a harder one because the variety is higher.

In Table 7, we included the results obtained by the existing solutions for the two protocols proposed in the second version of the NTU RGB+D dataset [8]. Even if the model proposed by us has a small number of parameters compared to such a varied dataset, which contains 120 classes, the performances obtained are satisfactory. For these protocols, there are two models for which the size is smaller, but the performances are better. The models are the same as in the case of the Cross-Subject (v1—60) and Cross-View protocols. For the Cross-Setups protocol, the model proposed by us achieves a slightly better performance than PA-ResGCN-N51.

### 4.5. Discussion

In this section, we analyze in detail the results obtained by the proposed model from the perspective of accuracy but also from the perspective of inference time. The role of these experiments is to demonstrate that the small inference time obtained for the model proposed by us, and the accuracy comparable to that of the other proposed models, makes our approach a suitable one for real-time running scenarios.

In Table 8, we included an analysis of the experimental results achieved by the proposed model for the Cross-Subject test protocol. We obtained these results by changing the number of epochs used in the training process. Based on them, we highlighted the fact that the proposed model does not require a large number of epochs for training. As can be seen, there is no difference between the Top 1 accuracy obtained for 100 epochs and the one obtained for 70. Moreover, the difference between the Top 5 accuracy for these two training configurations is insignificant. Due to the small number of parameters and the transformations proposed in this approach, it is possible to obtain an accuracy of 82.73% using only 10 epochs. In other words, using a larger number of training epochs helps the model distinguish between similar classes for which it predicts close probabilities. Moreover, we can see that by introducing augmentation transforms and using a Dropout-type layer with a probability of 0.3, we managed to obtain a model with a good generalization power that does not overfit. This observation is certified by the results obtained for 100 epochs which are not weaker than those for 70.

The proposed model obtains a good performance from the perspective of the Top 5 metrics. Therefore, we chose to perform an analysis of the results to understand why this considerable difference occurs between the performances for the Top 1 and those for the Top 5. In this sense, we determined the classes for which the proposed model obtains the best results. We were also interested to discover for which classes the model achieves the worst performance. We wanted to check what happens with the model prediction for the actions with the lowest performances.

**Figure 7:**
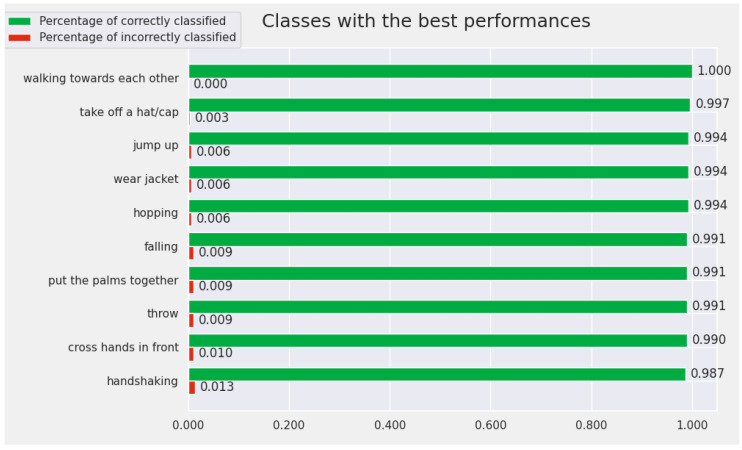
The Top 10 best-recognized classes by the proposed model (from the perspective of Cross-View protocol).

Figure 7 contains the ploy with the Top 10 best-recognized actions by the proposed model for the Cross-View protocol. The classes for which the convolutional model achieves the best performance for the Cross-View protocol are: *walking toward each other*, *take off a hat/cap*, *jump up*, *wear jacket*, *hopping*, *falling*, *put the palms together*, *throw*, *cross hands in front*, and *handshaking*. We highlighted, in Figure 7, the accuracy obtained by the model for these classes. For all 10 classes, the accuracy is over 98%.

**Figure 8:**
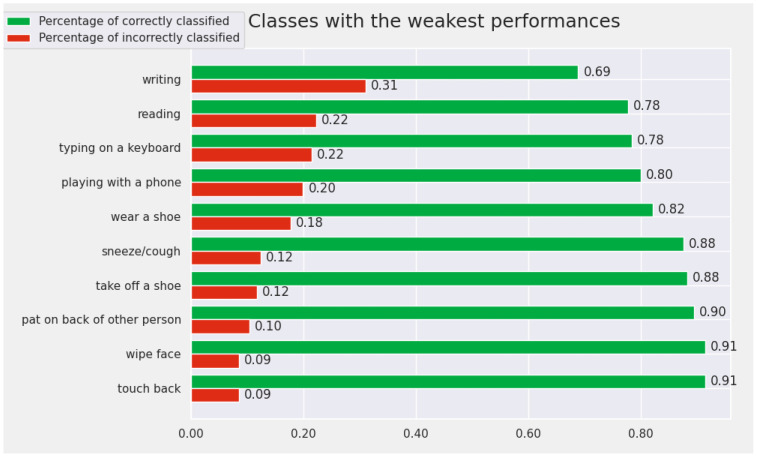
The Top 10 worst-recognized classes by the proposed model (from the perspective of Cross-View protocol).

At the opposite pole, the 10 classes for which the model achieves the worst results are: *writing*, *reading*, *typing on a keyboard*, *playing with a phone*, *wear a shoe*, *sneeze/cough*, *take off a shoe*, *pat on back of other person*, *wipe face*, and *touch back*. In this subset of actions, there are very similar pairs of actions for which it is difficult even for humans to differentiate between them. For example, the pair “writing” and “reading” contains very similar samples that are difficult to differentiate by visualizing only the human skeleton. We included the results for the weakest actions from the perspective of the Cross-View protocol in Figure 8.

**Figure 9:**
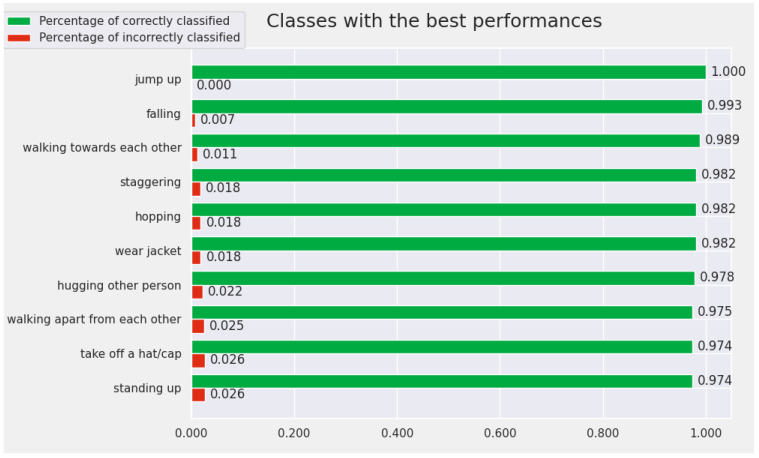
The Top 10 best-recognized classes by the proposed model (from the perspective of Cross-Subject protocol).

**Figure 10:**
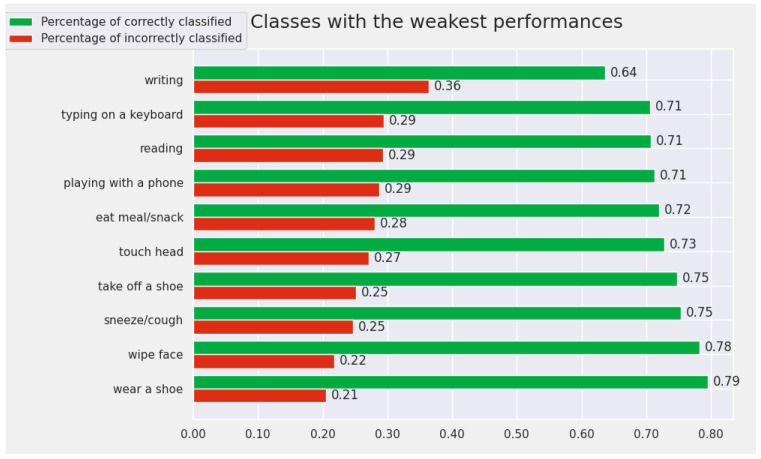
The Top 10 worst-recognized classes by the proposed model (from the perspective of Cross-Subject protocol).

In Figure 9 and Figure 10, we present similar plots for the Cross-Subject test protocol. This protocol is more difficult, and it reflected this in the experimental results. For this situation, only two actions have an accuracy greater than 99%.

**Figure 11:**
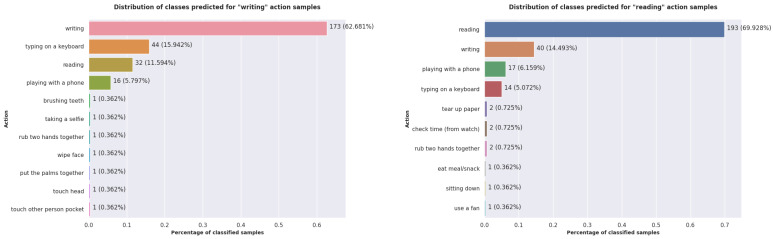
The distribution of the classes predicted by the proposed model for the *writing* and *reading* actions when using the Cross-Subject protocol.

We analyzed what happens with the prediction of the proposed model for the classes with the lowest performances: *reading* and *writing*. In Figure 11, we included the distribution of the predicted classes for the samples belonging to these two actions from the Cross-Subject protocol test set. For both actions, our model classified the most samples as one of the following classes: *writing*, *reading*, *typing on a keyboard*, and *playing with a phone*. When using the skeleton representation, these actions are difficult to differentiate, even for humans. It is important to note that very few samples exist for which the model predicts a class different from these similar actions. Humans performed these similar actions using their hands. This observation demonstrates that the model manages to correctly identify the interest part of the skeleton and somehow groups the similar actions according to this aspect.

**Figure 12:**
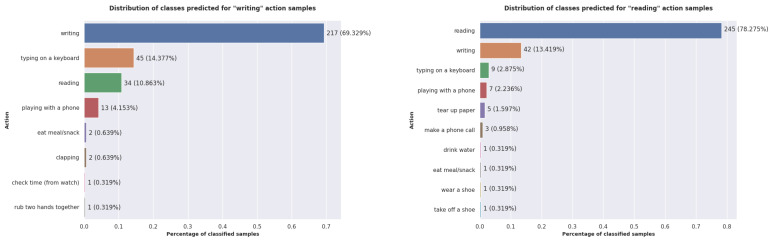
The distribution of the classes predicted by the proposed model for the *writing* and *reading* actions when using the Cross-View protocol.

To verify this assumption, we repeated the experiment for the Cross-View protocol and presented the obtained plots in Figure 12. The results obtained for this protocol confirm the previous observations, because this time, the predicted majority classes are the same four similar actions. For the reading action, the convolutional model predicts, for a significant number of samples, the classes *tear up paper* and *make a phone call*. Moreover, these actions are ones in which the most relevant part of the skeleton is the hand.

To highlight how difficult it is for people to differentiate between similar actions by analyzing only the skeleton representation, we chose five samples for the *writing* action and five samples for the *reading* action. Starting from these, we generated a video with plots of the joint coordinates for each sample. A group of 20 people analyzed these videos, and Table 9 presents the obtained results. The proposed model correctly recognizes four of the five samples for each action. For the four samples of the *reading* action correctly classified by the model, we have almost 100% confidence. On the other hand, we cannot say the same thing if we refer to the classifications performed by people. For a single sample, all 20 subjects who participated in the study correctly identified the action. This aspect proves that it is difficult for humans to visually differentiate between similar actions when only skeletal data are available. In the case of the *writing* action, the confidence predicted by our model is lower. Only for two of the four correctly classified samples does the confidence exceed 90%. We notice that there are samples for which there are human subjects who do not correctly identify the *writing* action.

**Figure 13:**
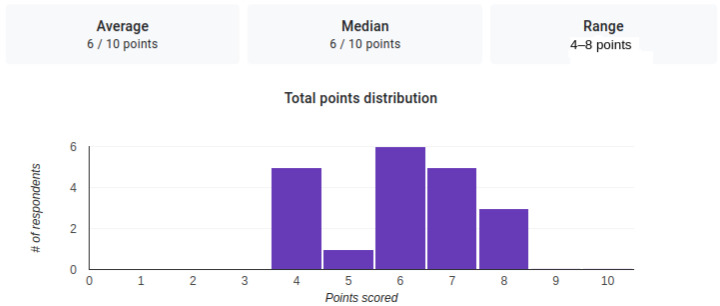
The distribution of the scores obtained by the 20 subjects for the correct identification of the actions.

In Figure 13, we included a plot that highlights the scores obtained by the 20 subjects in the study, conducted to discover how difficult it is for a person to distinguish between two similar actions when we use only skeletal data. As the average of the obtained scores is 6, we can conclude that it is a difficult operation for a person.

We wanted to evaluate the proposed model from the perspective of the inference speed. For this, we selected as a metric the number of sequences processed in one second on a GPU. In Table 10, we presented the results obtained for the proposed neural network and for the other two methods that contained a model with a smaller size, but which achieved better performances. To obtain these new results, we ran the evaluation procedure for the Cross-Subject protocol 100 times and averaged the speed. To perform the experiments, we used two NVIDIA A100 Tensor Core GPUs. Our convolutional model has the highest inference speed.

## 5. Conclusions

In this paper, we proposed a pipeline that can be used to solve the human action recognition problem. This pipeline includes three components: a module that augments the data, a module to preprocess the data to determine the geometrical features, and a neural convolutional module that classifies these data. We demonstrated through the obtained experimental results that the proposed pipeline achieves results comparable to the rest of the existing methods but requires a lower training time and has a better inference speed.

From the perspective of the experimental results, we can conclude that the proposed solution based on different convolutional layers is suitable for the problem of human action recognition using skeletal data. We recommend this approach both from the perspective of accuracy and from the perspective of a short inference time. The last aspect makes this very useful in solutions running in real-time scenarios. The augmentation stage applied in the proposed pipeline helps the solution to become robust by simulating the existence of data collected using different cameras. This aspect makes the proposed solution suitable for applications that use mobile sensors to collect data to identify human actions of interest. One such field that requires robust solutions that can analyze data collected during movement is that of social robots. Moreover, for this field, it is important that the neural network used requires limited hardware resources and is able to make fast predictions. It is important to note that the experimental results prove the possibility of combining multiple types of convolutional layers to develop a robust classifier with a high inference speed.

The problem of recognizing human actions has vast practical applicability, as emphasized by Kong and Fu in [29]. Some of the main fields in which the researchers included the specialized approaches in human action recognition are video retrieval, visual surveillance, entertainment, human–robot interaction, healthcare, and autonomous driving vehicles. Some of this practical applicability requires to correlate the model prediction with high-level features related to the context in which the action is performed. As our approach is focused on low-level features that describe the action at the kinematic level, one of the future research directions could be the development of methods that take into account the information that defines the context.

From the analysis of the results for each class, we discovered that the proposed model achieved weak results only for similar actions. Further development may consider adding additional features extracted from RGB images to solve this issue. Because the amount of information may be too large, we can consider cropping each image based on the coordinates of the skeleton.

Another possible direction that we can explore in further developments is to replace the use of a single classifier with a set of classifiers. In this approach, we need to propose a module for fusion predictions.

## Figures and Tables

**Table 1:** Performances obtained depending on the interval used to choose the random angle for rotation.

Method	θ	Top 1	Top 5
**Fast Convolutional** **(200 frames, dropout = 0.3, theta = 0.3)**	0.3	89.37	98.25
Fast Convolutional (200 frames, dropout = 0.3, theta = 0.3)	0.5	88.55	98.18
Fast Convolutional (200 frames, dropout = 0.3, theta = 0.3)	0.78	88.34	98.10

**Table 2:** Performances obtained depending on the augmentation transforms and the probability of the Dropout layer, where signifies the application of the transformation and ✗ indicates that we not included operation in the augmentation stage.

Uniform Sample	Random Move	Random Rotation	Dropout	Cross-Subject Accuracy (%)	Speed $\frac{\mathrm{No}.\;\mathrm{of}\;\mathrm{Sequences}}{\mathrm{Sec}\mathrm{ond}\;\times \;\mathrm{GPU}}$
200	✓	✗	0.3	88.31	246.85
200	✓	✓	0.5	88.82	241.74
200	✓	✓	✗	89.28	241.09
100	✓	✓	0.3	88.79	303.43
64	✓	✓	0.4	87.97	349.37
✗	✗	✗	0.3	89.14	174.85

**Table 3:** Performances obtained depending on the size of the kernels.

ResGCN		TCN		Cross-Subject Accuracy (%)	Speed $\frac{\mathrm{No}.\;\mathrm{of}\;\mathrm{Sequences}}{\mathrm{Sec}\mathrm{ond}\;\times \;\mathrm{GPU}}$
Temporal	Spatial	Temporal	Spatial		
9	2	9	1	88.95	240.06
9	2	11	1	89.36	235.37
11	3	5	1	89.43	228.33
9	3	5	1	89.22	230.38
9	2	5	3	89.27	240.29

**Table 4:** Comparative results from the perspective of accuracy for the proposed test protocols in the first version of the NTU RGB+D dataset [7].

Method Name	Model Size (M)	Cross-Subject (NTU v1—60) Accuracy (%)	Cross-View (NTU v1—60) Accuracy (%)
ST-GCN [13]	3.1	81.5	88.3
SR-TSL [19]	19.07	84.8	92.4
RA-GCN [20]	6.21	85.9	93.5
GR-GCN [21]	–	87.5	94.3
AS-GCN [12]	6.99	86.8	94.2
2s-AGCN [11]	6.94	88.5	95.1
AGC-LSTM [22]	22.89	89.2	95.0
DGNN [23]	26.24	89.9	96.1
SGN [24]	1.8	89.0	94.5
PL-GCN [25]	20.7	89.2	95.0
NAS-GCN [26]	6.57	89.4	95.7
ResGCN-N51 (Bottleneck) [9]	0.77	89.1	93.5
ResGCN-B19 (Basic) [9]	3.26	90.0	94.8
DSTA-Net [27]	–	91.5	96.4
PA-ResGCN-N51 [9]	1.14	90.3	95.6
MS-G3D Net [28]	3.2	91.5	96.2
PA-ResGCN-B19 [9]	3.64	90.9	96.0
CTR-GCN [5]	1.46	92.4	96.8
ResGCN-TCN [18]	5.13	88.68	94.04
ResGCN-LSTM [18]	2.15	85.01	92.3
Fast Convolutional (ours)	1.58	89.37	94.86

**Table 5:** Performances obtained by the convolutional model for the NTU RGB+D dataset [7] from the perspective of Top 1 and Top 5 accuracy.

Method Name	Type	Cross-Subject (NTU RGB+D [7]) Accuracy (%)	Cross-View (NTU RGB+D [7]) Accuracy (%)
Fast Convolutional (ours)	Top 1	89.37	94.86
Fast Convolutional (ours)	Top 5	98.17	99.39

**Table 6:** Performances obtained by the convolutional model for the NTU RGB+D dataset [8] from the perspective of Top 1 and Top 5 accuracy.

Method Name	Type	Cross-Subject 120 (NTU RGB+D [8]) Accuracy (%)	Cross-Setup (NTU RGB+D [8]) Accuracy (%)
Fast Convolutional (ours)	Top 1	85.82	87.14
Fast Convolutional (ours)	Top 5	97.41	97.24

**Table 7:** Comparative results from the perspective of accuracy for the proposed test protocols in the second version of the NTU RGB+D dataset [8].

Method Name	Model Size (M)	Cross-Subject (NTU v2—120) Accuracy (%)	Cross-Setups (NTU v2—120) Accuracy (%)
ST-GCN [13]	3.1	70.7	73.2
RA-GCN [20]	6.21	74.6	75.3
AS-GCN [12]	6.99	77.9	78.5
2s-AGCN [11]	6.94	82.5	84.2
SGN [24]	1.8	79.2	81.5
ResGCN-N51 (Bottleneck) [9]	0.77	84.0	84.2
ResGCN-B19 (Basic) [9]	3.26	85.2	85.7
DSTA-Net [27]	–	86.6	89
PA-ResGCN-N51 [9]	1.14	86.6	87.1
MS-G3D Net [28]	3.2	86.9	88.4
PA-ResGCN-B19 [9]	3.64	87.3	88.3
CTR-GCN [5]	1.46	88.9	90.6
ResGCN-TCN [18]	5.13	84.4	84.6
ResGCN-LSTM [18]	2.15	79.93	81.28
Fast Convolutional (ours)	1.58	85.82	87.14

**Table 8:** Performances achieved by the proposed model for the Cross-Subject protocol (v1—60) depending on the number of epochs set for training.

Method	Total Number of Epochs	Top 1	Top 5	Best Epoch
Fast Convolutional (200 frames, dropout = 0.3, theta = 0.3)	100	89.37	98.25	97
Fast Convolutional (200 frames, dropout = 0.3, theta = 0.3)	70	89.37	98.17	66
Fast Convolutional (200 frames, dropout = 0.3, theta = 0.3)	50	89.05	98.20	49
Fast Convolutional (200 frames, dropout = 0.3, theta = 0.3)	30	87.65	98.09	29
Fast Convolutional (200 frames, dropout = 0.3, theta = 0.3)	10	82.73	97.13	10

**Table 9:** Statistics for how people perceive similar actions by analyzing a graphical representation of skeletal data.

Sample	No. of Responses Reading	No. of Responses Writing	Percentage	Model Prediction
Reading 1	3	17	15%	Reading (100%)
Reading 2	13	7	65%	Reading (99.9%)
Reading 3	20	0	100%	Reading (100%)
Reading 4	15	5	75%	Eat meal (93.4%)
Reading 5	7	13	35%	Reading (100%)
Writing 1	10	10	50%	Writing (95.8%)
Writing 2	6	14	70%	Type on keyboard (99.7%)
Writing 3	12	8	40%	Writing (57.3%)
Writing 4	3	17	85%	Writing (47.1%)
Writing 5	7	13	65%	Writing (99.5%)

**Table 10:** Comparative performances from the perspective of computability (processing speed and number of parameters).

Method	Speed $\frac{\mathrm{No}.\;\mathrm{of}\;\mathrm{Sequences}}{\left(\mathrm{Sec}\mathrm{ond}\;\times \;\mathrm{GPU}\right)}$	Cross-Subject (NTU RGB+D v1) Accuracy (%)	Model Size
Fast Convolutional (ours)	233.23	89.37	1.58
PA-ResGCN-N51	180.80	90.3	1.14
CTR-GCN	115.64	92.4	1.46

## Data Availability

Not applicable.

## Abbreviations

The following abbreviations are used in this manuscript:

TCN	Temporal Convolutional Network
GCN	Graph Convolutional Network
HAR	Human Action Recognition
AmI	Ambient Intelligent
RNN	Recurrent Neural Network
CTR-GC	Channel-wise Topology-Refinement Graph Convolution
ResGCN	Residual Graph Convolutional Network
SGD	Stochastic Gradient Descent
GPU	Graphics Processing Unit
